# Nanogap enhancement of the refractometric sensitivity at quasi-bound states in the continuum in all-dielectric metasurfaces

**DOI:** 10.1515/nanoph-2022-0565

**Published:** 2023-01-03

**Authors:** Keisuke Watanabe, Masanobu Iwanaga

**Affiliations:** International Center for Young Scientists (ICYS), National Institute for Materials Science (NIMS), 1-1 Namiki, Tsukuba, Ibaraki 305-0044, Japan; Research Center for Functional Materials, National Institute for Materials Science (NIMS), 1-1 Namiki, Tsukuba, Ibaraki 305-0044, Japan

**Keywords:** all-dielectric, biosensors, bound states in the continuum, metasurfaces, nanogaps

## Abstract

All-dielectric metasurfaces have great potential as highly sensitive refractometric sensors relying on their spectral shifts because of an extensive range of design flexibilities and their smaller absorption losses than plasmonic platforms. However, simultaneously realizing both high quality (*Q*) factors and the large interplay of light with external medium in such photonic sensors remains one of the key challenges for their better performance. This study proposes silicon block metasurfaces with nanogaps to overcome this challenge based on quasi-bound states in the continuum (BICs). We show that the metasurface has two quasi-BIC modes—magnetic dipole (MD) and electric quadrupole (EQ)—and their electric fields experience large enhancement at the ∼30 nm nanogap regions. Consequently, introducing nanogaps into the metasurfaces increases the environmental refractive index sensitivity by up to 2.7 times in the MD mode while keeping the high *Q* factors and achieves the figure-of-merit (FOM) of 239. In addition, we show that the appropriate selection of the amount of asymmetry is needed under the trade-off between the FOM and spectral signal-to-noise ratio, which provides design guidelines for highly sensitive biosensors based on quasi-BICs.

## 1 Introduction

Enhancing light–matter interactions at the nanoscale is important for highly sensitive sensing applications. Optical cavities such as photonic crystals [1, 2], plasmonic structures [3, 4], and the hybrid configurations [5, 6] have been developed for this purpose using their ability of strong electromagnetic field confinement. Recently, high refractive index dielectric metasurfaces have attracted significant attention as new strategies because of the diverse tunability of their optical properties based on their Mie resonances, small material absorption losses, and low-cost fabrication [7]. Hence, all-dielectric metasurfaces have great potential for versatile applications [8–11]. As metasurfaces derive their function from the individual shape of the unit structure, a wide variety of design flexibilities exist as building blocks depending on the application. Moreover, a collective response at a singularity, called bound state in the continuum (BIC), in the dielectric array offers several possibilities as functional materials [12–15]. BICs are specific states that are localized with no leakage and zero linewidth [14] inside the radiation continuum. The out-of-plane radiative pathway is completely suppressed in an infinitely large nanostructure in the lateral direction owing to the symmetry mismatch between the mode profiles inside the structure and those of the external continuum [16]. Therefore, optical devices with infinite quality (*Q*) factors can be realized in an ideal case. This type of BIC is known as symmetry-protected BIC, where the sharp peaks are experimentally accessible from the free space by breaking the symmetry of the unit structure (quasi-BIC) [17]. The sensing applications reported so far include high refractive index sensitivity [18], biotin-streptavidin bioassay [19], detection of breast cancer extracellular vesicles [20], enhanced infrared absorption [21], surface-enhanced Raman scattering [22], and chiral molecule detection [23]. However, one of the key challenges in developing more sensitive refractometric sensors based on their spectral shifts is to increase the interaction between light and the surrounding medium while maintaining their sharp resonances (= high *Q* factors). The large interaction between light and external medium increases the environmental refractive index sensitivity *S*_env_, which is defined as *S*_env_ = Δ*λ*/Δ*n* (nm/RIU), where Δ*λ* denotes the wavelength shift due to increasing environmental refractive index Δ*n*. Therefore, it is important to well optimize the metasurface structure and increase a commonly used figure of merit (FOM), which is given by FOM = *S*_env_/FWHM, where FWHM (nm) corresponds to the full width at half maximum of the resonance peak [18]. Compared with plasmonic resonators, which typically suffer from large FWHM owing to their intrinsic losses, quasi-BIC modes contribute significantly to the reduction of FWHM. On the contrary, little work has devoted effort to increase the refractive index sensitivity *S*_env_ of dielectric metasurfaces by engineering their unit nanostructures. Simulations in the literature have predicted that employing nanogaps in the quasi-BIC metasurfaces increases the refractive index sensitivity [24–27]. However, no experimental work incorporating nanogaps was performed except for a split-ring resonator [28] whose FOM of the Fano resonance was only 37.

In this work, we demonstrate a high FOM using all-dielectric metasurfaces with nanogaps in each unit cell, where electromagnetic fields are concentrated into the surrounding medium. We show that sharp resonances at quasi-BICs in the dielectric array provide both high *Q* factor and large bulk refractive index sensitivity. In the experiment, the metasurfaces are fabricated using silicon-on-quartz wafers. Their optical properties are then characterized by angle-resolved transmittance measurements and reflectance measurements at normal incidence using a home-built setup. This study experimentally demonstrates the FOM of 239, which is among the highest in all-dielectric metasurfaces. Furthermore, we note the importance of the appropriate selection of the asymmetry in refractometric sensors based on quasi-BIC metasurfaces by considering the trade-off relationship between the FOM and signal-to-noise ratio (SNR). These findings will be useful in applications requiring large light–matter interactions, especially in sensing.

## 2 Results and discussions

**Figure 1:**
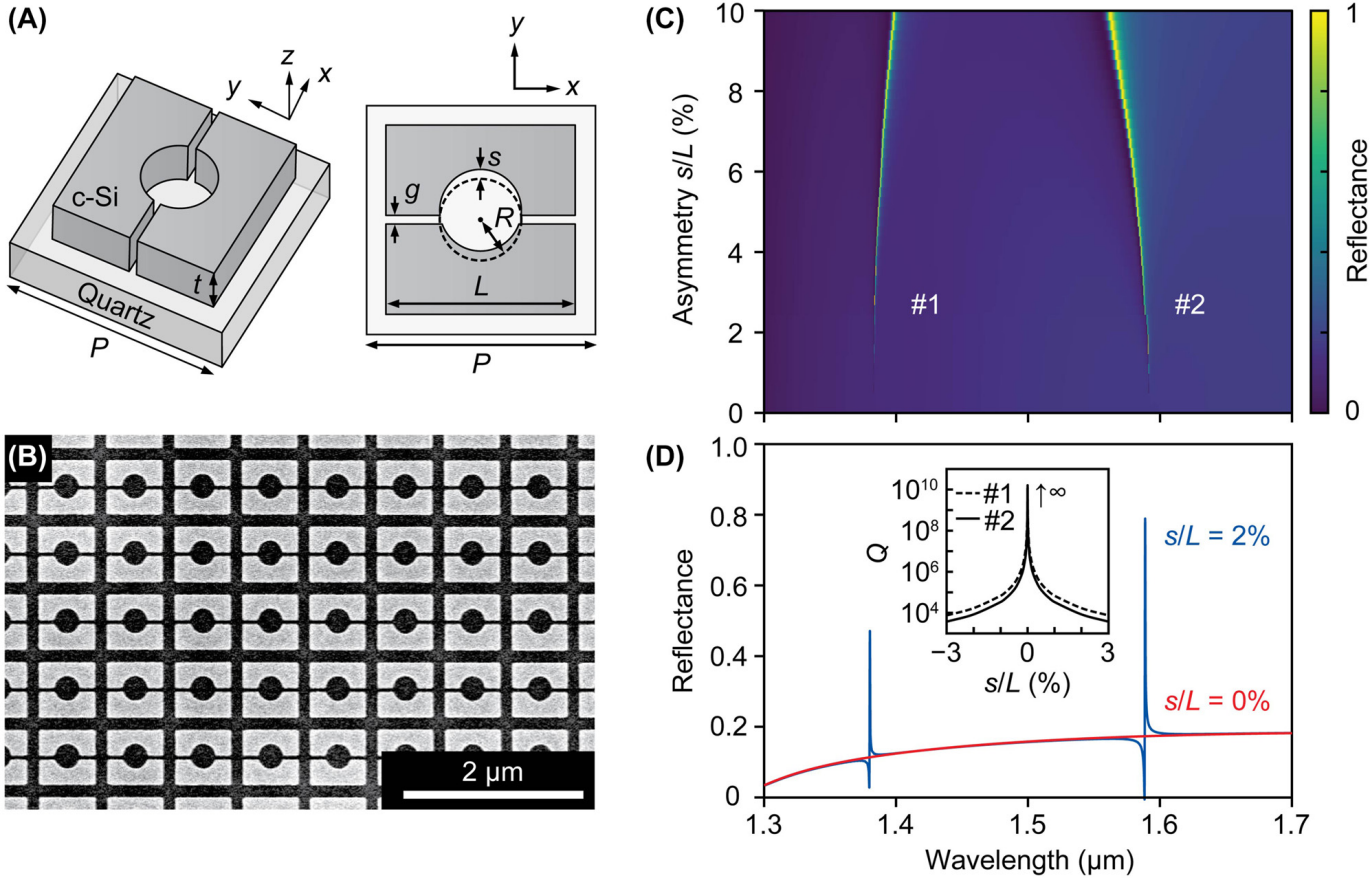
Structure and basic characteristics of all-dielectric metasurfaces with nanogaps. (A) Schematic of the unit cell and the structural parameters depicted in three dimensions (left) and the top view (right). (B) SEM image of a fabricated metasurface with the measured gap size of approximately 33 nm. (C) Simulated contour map of the reflectance spectra for the metasurface with different asymmetries *s*/*L* excited by a plane wave along the *x* direction (*P* = 750 nm, *L* = 580 nm, *t* = 200 nm, *R* = 135 nm, *g* = 30 nm), where the superstrate is assumed to be water (*n* = 1.33). The two quasi-BIC modes are denoted as #1 and #2. (D) Simulated reflectance spectra of the metasurfaces with (*s*/*L* = 2%, blue curve) and without (*s*/*L* = 0%, red curve) broken symmetries. Inset shows the calculated *Q* factors of the two quasi-BIC modes.

Figure 1A shows the proposed metasurface structure. Crystalline silicon (c-Si) blocks with a height and a side length of *t* = 200 nm and *L* = 580 nm, respectively, are arranged in a square lattice with a period of *P* = 750 nm on a quartz substrate. In each unit cell, nanogaps with a width of *g* are employed parallel to the *x* direction and an airhole with a radius of *R* = 135 nm is perforated with a shift *s* from the center in the *y* direction to provide asymmetry in the structure. The in-plane asymmetry is characterized by *s*/*L*. The silicon block metasurfaces were fabricated using electron beam (EB) lithography on silicon-on-quartz (SOQ) wafers and the dry-etching process of silicon without using metal deposition processes (see Methods for more details). Figure 1B shows a representative image of a fabricated sample captured by a field-emission scanning electron microscopy (SEM) (S-4800, Hitachi High-Tech). The average size of nanogaps was 33.0 ± 4.9 nm, which is considered to be the minimum size that can be fabricated in our fabrication processes. The electromagnetic simulation based on a time domain electromagnetic solver (Ansys Lumerical FDTD) reveals that two Fano resonances originating from quasi-BICs appear when the *x*-polarized normal incident plane wave is coupled to the metasurface with broken symmetry to *y* directions, as shown in Figure 1C and D. Asymmetry to *y* direction results in the deformation of the displacement current loops inside silicon blocks, whose net electric components have *x* direction, opening the radiative pathway to the out-of-plane radiation [26, 29]. Therefore, coupling the two quasi-BIC modes from the free space is only possible with a linearly polarized incident wave along the *x* direction. With a *y*-polarized plane wave, by contrast, light is decoupled from the incident plane wave (see Figure S1 Supporting Information for the polarization dependence) due to symmetry incompatibility [30]. We note that incorporating nanogaps parallel to the *x* direction does not affect the net electric components in the *x* direction. Therefore, the symmetry-protected bound state is unperturbed; the two quasi-BIC modes are protected by in-plane symmetry. Figure 1C and D also shows that the degree of leakage to the free space depends on the amount of asymmetry *s*/*L*. The *Q* factors decreases exponentially with increasing *s*/*L*, and the reflectance increases concurrently.

**Figure 2:**
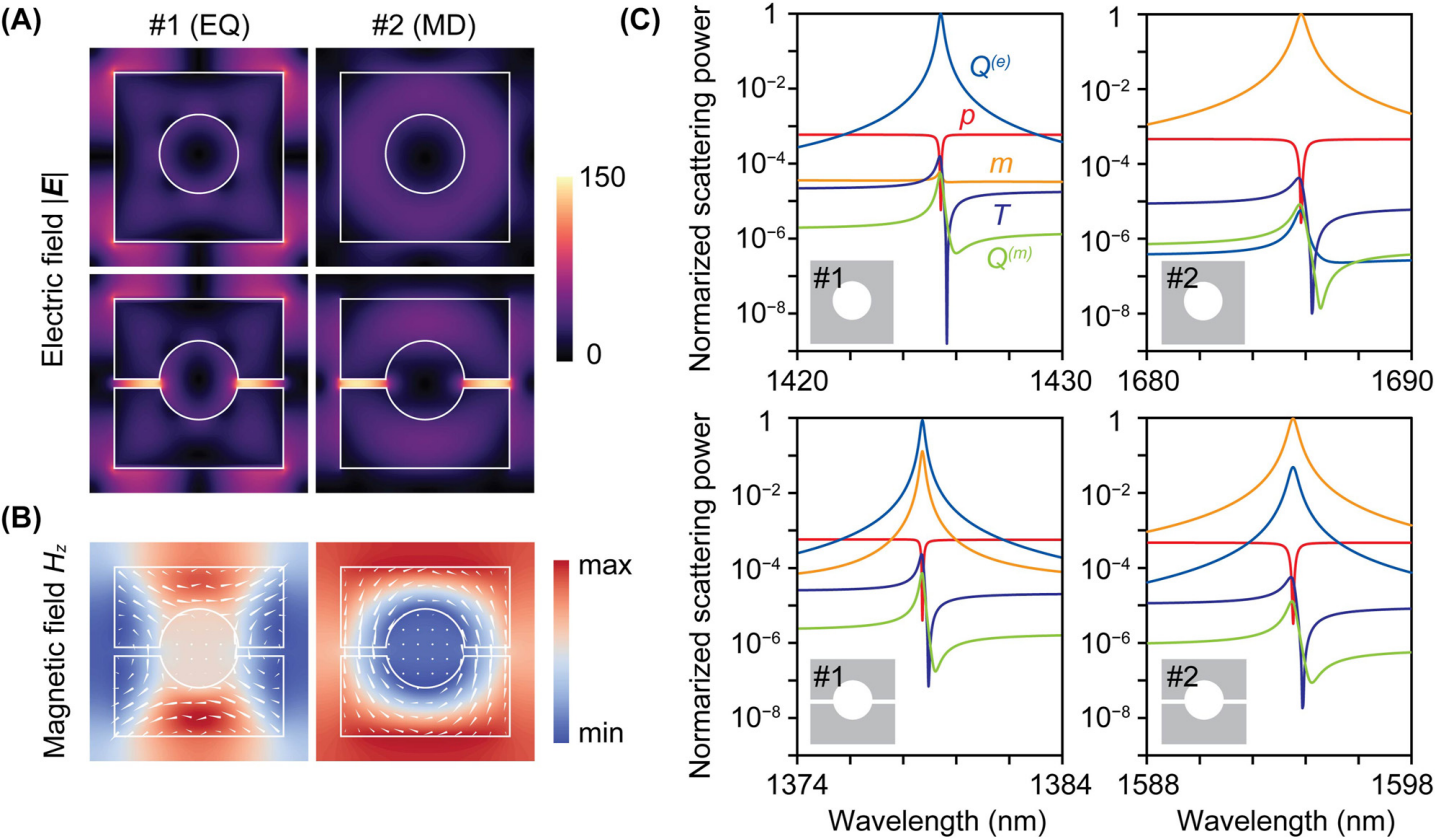
Field distributions and the origin of the two quasi-BIC modes. (A) Simulated electric field | ***E*** | profiles for metasurfaces (*s*/*L* = 2%) without nanogaps (upper) and with nanogaps (lower) for mode #1 (left) and #2 (right), respectively. (B) Simulated *H*_*z*_ profiles for the metasurface with nanogaps. White arrows show the displacement current. (C) Multipole contributions in scattering powers obtained by the multipole decomposition in each resonance mode. The scattering powers are normalized to the sum of the components of all the multipoles. *p*: electric dipole; *T*: toroidal dipole; *m*: magnetic dipole; *Q* ^(*e*)^: electric quadrupole; *Q* ^(*m*)^: magnetic quadrupole.

Figure 2A shows the near-field mode profiles of the metasurface with and without nanogaps. The electric fields experience significant enhancement after introducing nanogaps (*g* = 30 nm) with the help of cavity resonances under quasi-BIC conditions as well as the large refractive index contrast between silicon and external medium (water) for both quasi-BIC modes (#1 and #2). The localization of electric fields originates from the large discontinuity in the *y* component of electric field *E*_*y*_ and the conservation of the displacement field between silicon and water, similar to slotted waveguides [31]. These results clearly indicate that the electric fields inside the nanogaps maximized at the quasi-BIC resonance wavelengths largely interact with the surrounding medium. These behaviors can also be understood from the displacement current superimposed on *H*_z_ profiles for the transverse electric (TE)-like mode (Figure 2B). Anti-parallel currents perpendicular to the nanogaps on either side (right and left) of the silicon block can be seen, which account for the nanogap enhancement of the fields. To be more specific, the displacement current flow for mode #1 is equivalent to anti-parallel two electric dipoles, indicating an electric quadrupole moment. Mode #2 has a circulating current along the hole, which can be regarded as a magnetic dipole moment. To validate the origin of the two peaks, multipole decomposition is conducted on the basis of the Cartesian coordinate system [32, 33]. We calculate the induced multipole moments using the equations given by Ref. [34] based on the displacement current density ***j*** (***r***) inside the unit structure as

(1) $$P=\frac{1}{i\omega }\int jd^{3}r,$$

(2) $$M=\frac{1}{2c}\int \left(r\times j\right)d^{3}r,$$

(3) $$T=\frac{1}{10c}\int \left[\left(r\cdot j\right)r-2r^{2}j\right]d^{3}r,$$

(4) $$Q_{\alpha ,\beta }^{\left(e\right)}=\frac{1}{2i\omega }\int \left[\left(r_{\alpha }j_{\beta }+r_{\beta }j_{\alpha }\right)-\frac{2}{3}\left(r\cdot j\right)\delta_{\alpha ,\beta }\right]d^{3}r,$$

(5) $$Q_{\alpha ,\beta }^{\left(m\right)}=\frac{1}{3c}\int \left[{\left(r\times j\right)}_{\alpha }r_{\beta }+{\left(r\times j\right)}_{\beta }r_{\alpha }\right]d^{3}r,$$

where *ω* denotes the angular frequency, *c* is the speed of light, and *α*, *β* = *x*, *y*, *z*. The ***P***, ***M***, ***T***, ***Q*** ^(*e*)^, and ***Q*** ^(*m*)^ correspond to the multipole moments of electric dipole (ED), magnetic dipole (MD), toroidal dipole (TD), electric quadrupole (EQ), and magnetic quadrupole (MQ), respectively. The scattering power contributing to the far-field response is then calculated by

(6) (6) $$\begin{array}{ll} I & =\frac{2\omega^{4}}{3c^{3}}{\left|P\right|}^{2}+\frac{2\omega^{4}}{3c^{3}}{\left|M\right|}^{2}+\frac{2\omega^{6}}{3c^{5}}{\left|T\right|}^{2}+\frac{\omega^{6}}{5c^{5}}\sum_{\alpha ,\beta }{\left|Q_{\alpha ,\beta }^{\left(e\right)}\right|}^{2}\\ & \;+\frac{\omega^{6}}{20c^{5}}\sum_{\alpha ,\beta }{\left|Q_{\alpha ,\beta }^{\left(m\right)}\right|}^{2}. \end{array}$$

The calculation results (Figure 2C) reveal that the dominant multipole components are EQ moment *Q* ^(*e*)^ and MD moment *m* for both with and without nanogaps. Specifically, the dominant multipoles (EQ and MD) are more than three orders of magnitude than other contributing multipoles without nanogaps. The EQ and MD are mixed with nanogaps; however, the dominant contribution of the EQ (MD) is still an order of magnitude higher for #1 (#2) than other multipole components. Therefore, we name #1 as EQ and #2 as MD in the following.

**Figure 3:**
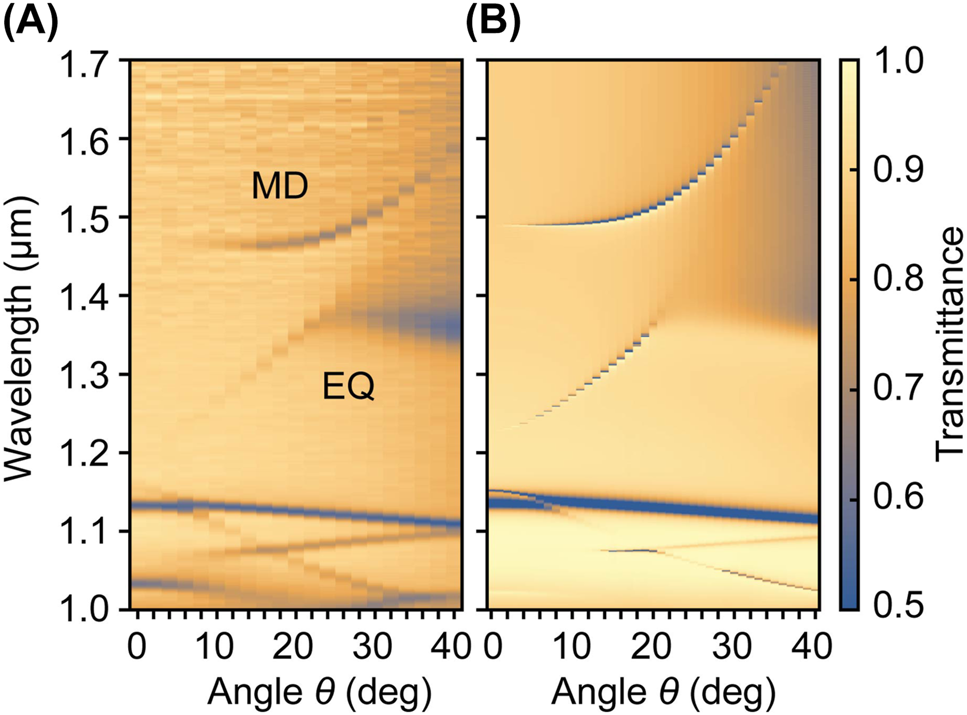
Angle-resolved transmittance measurements. (A) Transmittance spectra obtained by experiment and (B) FDTD simulation. In both cases, *s*-polarized light with a wave vector along the *y* direction was used to couple light into the metasurface without broken symmetry (*s*/*L* = 0%).

To experimentally verify the existence of EQ and MD modes, angle-resolved transmittance spectra were measured in the air using a metasurface without broken symmetry (*s*/*L* = 0%). We used a UV-VIS-NIR spectrometer with an incident-angle-resolving unit (V7200, JASCO). An *s*-polarized incident wave with the wave vector along the *y* direction was injected from the top of the metasurface for the coupling with the quasi-BIC modes, which correspond to the modes with TE-like polarization. Figure 3 compares the results obtained by experiment and finite-difference time-domain (FDTD) simulation, showing a good agreement. The net electric field component in the *x* direction is zero under normal incidence (*θ* = 0°), and the two quasi-BIC modes are decoupled from the incident plane wave due to the symmetry mismatch. Breaking the symmetry by deviating *θ* from zero opened the radiative channel; EQ and MD modes were observed in the experiment. For large angles of incidence, the quasi-BIC modes became more distinct, and the *Q* factors decreased, which agrees well with the explanation in the introductory part. We note that the large transmittance dips around 1.13 μm at *θ* = 0° are doubly degenerate modes [35] that have access to the external radiation field [30].

**Figure 4:**
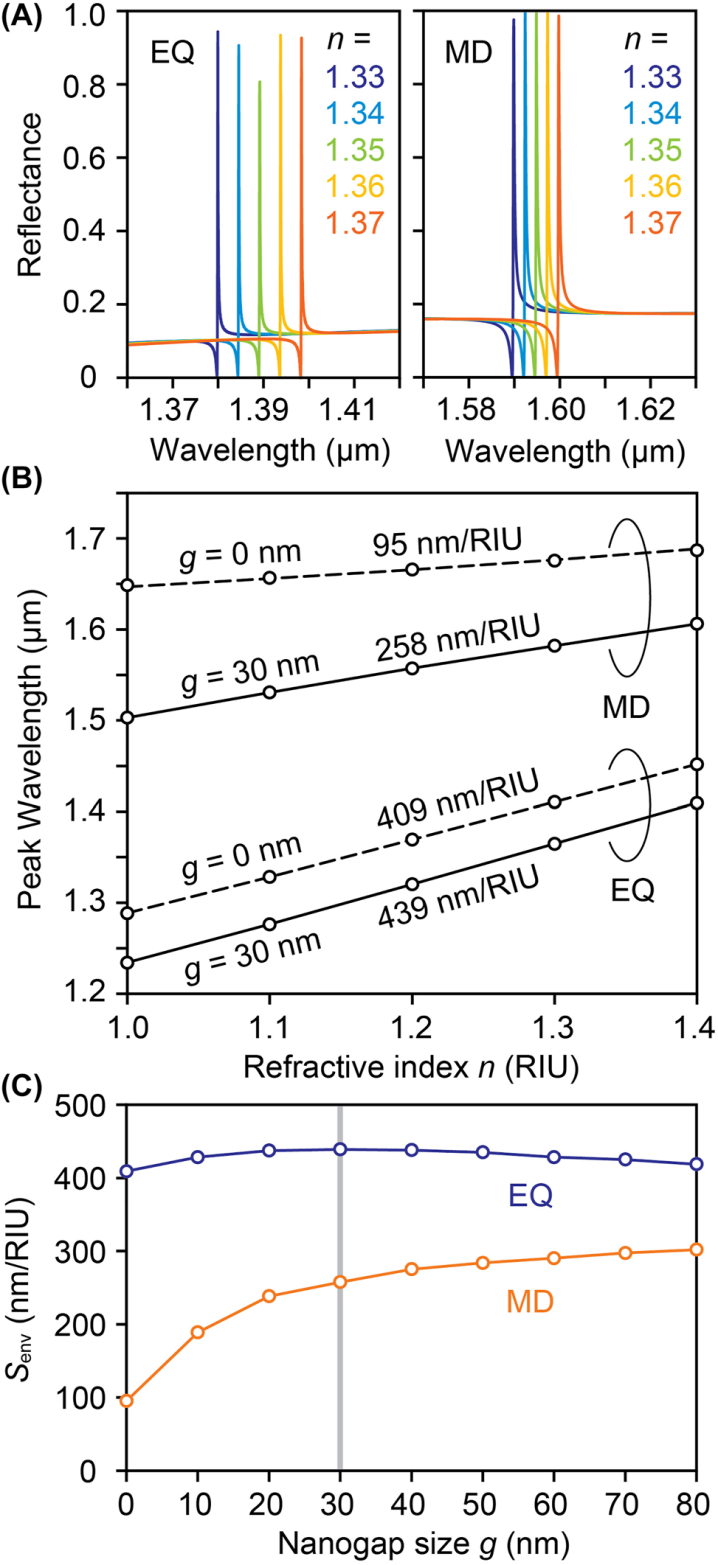
Simulated refractometric sensing results. (A) Resonance spectra of the metasurface (*s*/*L* = 2%) in different environmental refractive indices for EQ (left) and MD (right) modes. (B) The resonance peak positions as a function of the bulk refractive index. The refractometric sensitivities *S*_env_ are determined from the slope of the linear regression curve. (C) Calculated *S*_env_ as a function of nanogap size *g.* EQ and MD modes are shown with blue and orange open circles, respectively. Vertical line highlights the results when *g* = 30 nm.

We then simulate the resonance peak shifts at different environmental refractive indices and evaluate the refractometric index sensitivities for both EQ and MD modes. Figure 4A shows the resonance spectra of the metasurface in different environmental refractive indices for EQ and MD modes. The EQ mode has a larger wavelength shift than that of the MD mode, implying large refractive index sensitivity *S*_env_. The sensitivity *S*_env_ is determined from the slope of the linear regression of the resonance peak wavelength as a function of the environmental refractive index, as shown in Figure 4B. We obtain a sensitivity of 258 nm/RIU for the MD mode and a larger sensitivity of 439 nm/RIU for the EQ mode. Comparing both with and without nanogaps, the MD mode demonstrates 2.7 times higher sensitivity *S*_env_. However, the enhancement of the refractive index sensitivity for the EQ mode is much lower than that for the MD mode. These behaviors can be understood by the fact that a large part of the electric fields in the EQ mode is localized outside the silicon blocks even with nanogaps, as seen in the mode profiles in Figure 2C. Therefore, introducing nanogaps does not significantly change the overlapping of the electric fields with the outside medium and so does not increase the sensitivity. Figure 4C shows the dependence of the *S*_env_ on nanogap size *g*. The *S*_env_ of the MD mode is susceptible to the nanogap size and saturates around *g* = 30 nm. The *S*_env_ slightly increases with larger gaps; however, we set the target gap size as 30 nm to minimize the decrement of the *Q* factors due to the increased surface area.

At first glance, the EQ mode appears more favorable to the sensing because of the higher sensitivity than the MD mode. However, the *Q* factor of the EQ mode is more likely to be affected by fabrication imperfections. It has been known that the quasi-BIC modes are sensitive to fabrication imperfections primarily due to the scattering loss through the surface roughness of the silicon [36]. As a matter of fact, the fabricated metasurfaces in our experiment had local deformations and rounded corners where the electric components are mainly localized in the EQ mode and had lower *Q* factors (see Figure S2 and Figure S3 Supporting Information for the measured spectra). Therefore, the FOM of the EQ mode can be degraded even though the refractive index sensitivity is larger than that of the MD mode. This result is consistent with a study published last year, in which a higher order mode of the all-dielectric metasurface had large interaction with the surrounding medium and had large *S*_env_; however, the experimental *Q* factors were significantly lower than those in the simulation [19]. Therefore, we will only focus on the MD mode in the following. Because the MD mode is originally confined inside the silicon blocks and have a small overlap with the surrounding medium, introducing nanogaps exert a larger impact on the interaction between the localized electric fields inside nanogaps and the surrounding medium. Yet, the MD mode is robust to the fabrication imperfections and minimizes the reduction in the *Q* factors because the electric fields are mostly confined inside the sufficiently small nanogap regions.

**Figure 5:**
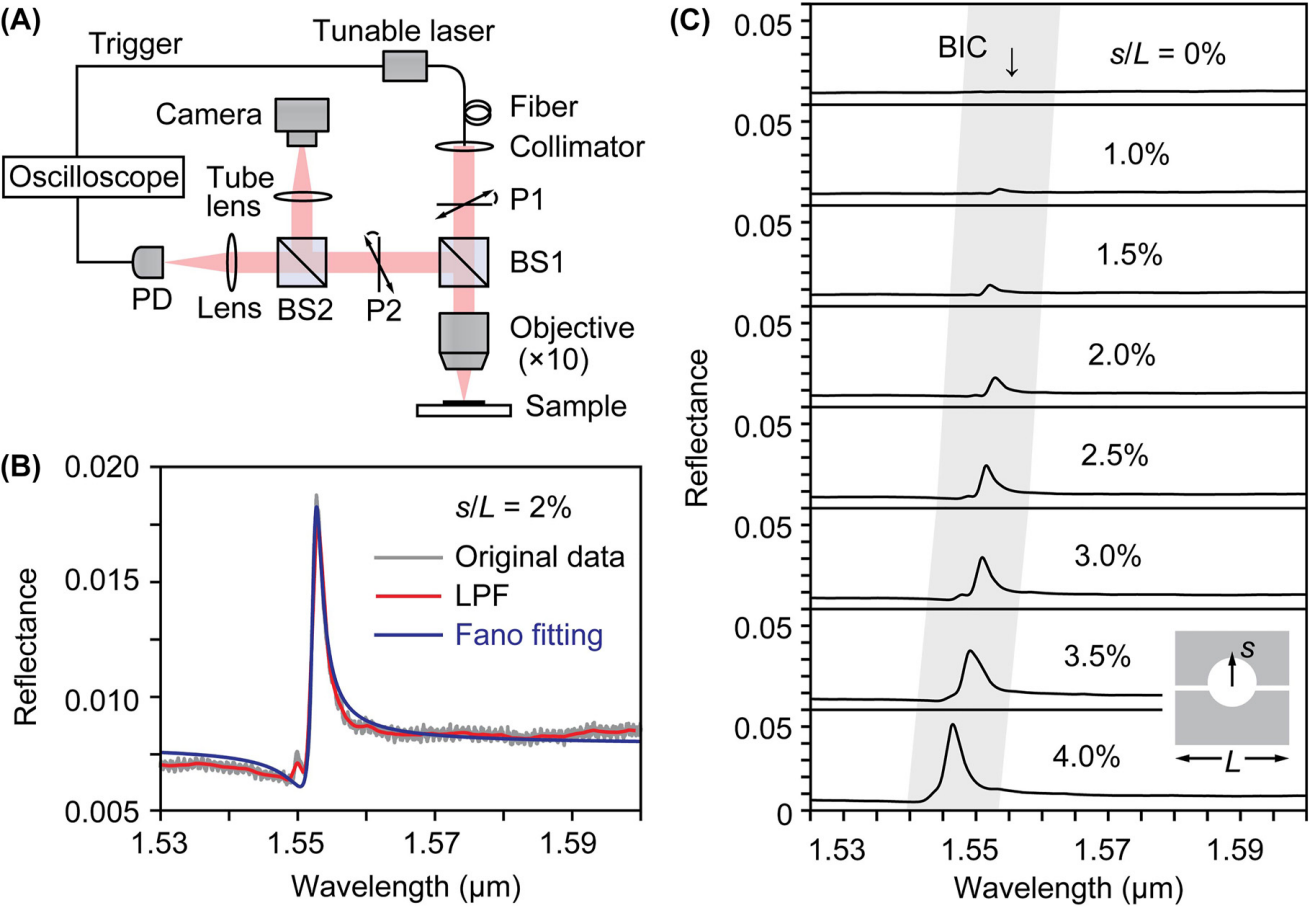
Reflectance measurements in water at normal incidence using a home-built setup. (A) Experimental setup for the reflectance measurement. P1 and P2: polarizers; BS1 and BS2: beam splitters; PD: photodiode. (B) Reflectance spectrum for the metasurface (*s*/*L* = 2%) with nanogaps measured in water. (C) Spectra of the MD mode with different asymmetries *s*/*L*. The vertical thick grey line represents guide to the eye.

Hereafter, reflectance spectra with higher wavelength resolution were measured using a home-built setup to characterize the sensing performance of the metasurfaces. Figure 5A depicts the experimental setup. The wavelength of the incident wave was swept by a tunable laser, and the reflection from the metasurfaces was acquired with a photodiode. The two polarizers were kept cross-polarized to minimize the background noise [37]. Figure 5B shows the measured reflectance spectrum for the MD mode in pure water. The unwanted interference noise generated from the transparent SOQ wafer was removed by software low-pass filtering. The reflectance spectrum showed a typical asymmetric Fano lineshape; it was fitted with the following function [38]:

(7) $$F\left(\delta \right)=A_{0}+F_{0}\frac{{\left(q+\delta \right)}^{2}}{1+\delta^{2}},$$

where *A*_0_ and *F*_0_ are the constant factors and *q* denotes the Fano asymmetry parameter. Furthermore, *δ* = (*ω* − *ω*_0_)/*γ,* where *ω*_0_ corresponds to the resonance peak frequency and *γ* represents the damping rate. The experimental *Q* factor (= *ω*_0_/2*γ*) was then evaluated as *Q* = 923 for the metasurface with the asymmetry *s*/*L* of 2%. The *Q* factor was significantly reduced compared to that obtained via simulation, which may be due not only to the surface roughness discussed above, but also the overtone absorption of water as well as infinite array configuration. Note that a small peak at ∼1.55 μm was possibly due to irregular drift or displacement of the airholes during the EB lithography, which is not included in the following discussion. Figure 5C shows the reflectance spectra of metasurfaces with different asymmetries *s*/*L*. The Fano resonances originating from the quasi-BIC became more apparent with increasing *s*/*L* due to the increased out-of-plane radiation. Simultaneously, the *Q* factors decreased, and the peak positions shifted to shorter wavelengths. Resonance peaks were not observed without asymmetry (*s*/*L* = 0%) because the out-of-plane radiation was completely prohibited. These behaviors clearly reflect the characteristics of the MD mode excited by *x*-polarized light.

**Figure 6:**
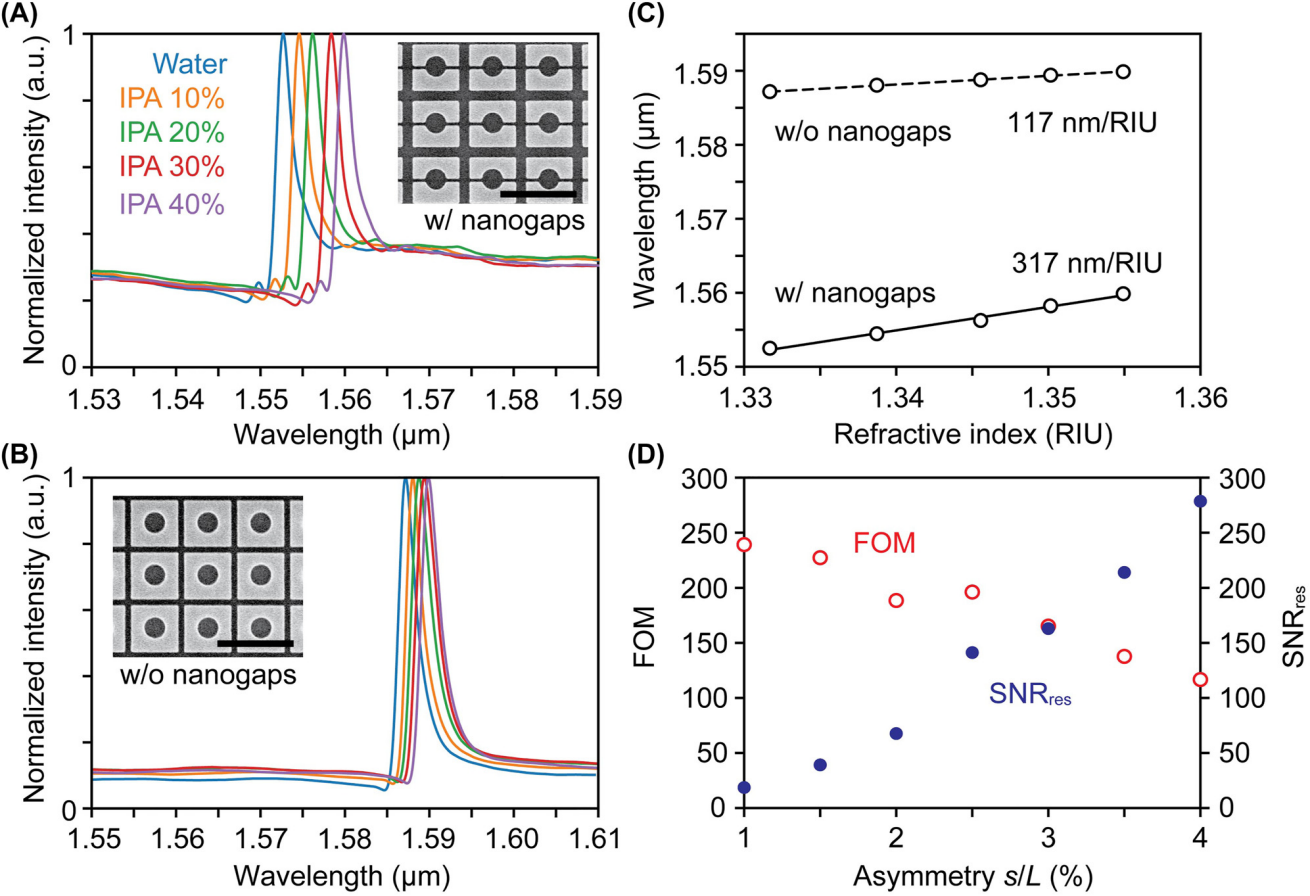
Measurement results of the refractive index sensitivity. (A), (B) Normalized reflectance spectra measured in different concentrations of IPA for the metasurfaces (*s*/*L* = 2%) with and without nanogaps, respectively. Insets show the SEM images with the scale bars of 1 μm. (C) Resonance peak shift of the metasurfaces with (solid line) and without (dashed line) nanogaps as a function of the bulk refractive index. (D) FOM (red open circles) and SNR_res_ (blue closed circles) with different asymmetries *s*/*L* for the metasurfaces with nanogaps.

Next, a metasurface with broken symmetry *s*/*L* of 2%, which maintained a large *Q* factor and peak intensity, was set to a dimethylpolysiloxane (PDMS) microfluidic channel. The liquid solutions with different refractive indices prepared by mixing pure water and isopropyl alcohol (IPA) were injected. Here, their refractive indices were pre-checked by a refractometer (PAL-RI, Atago) for reference. Figure 6A and B shows the measured reflectance spectra. The peak positions were redshifted with increasing liquid refractive indices. Note that the size of the unit cell was slightly shrunk (*P* = 690 nm) for the metasurface without nanogaps such that the peak position resided within the sweep wavelength range of the tunable laser. In this case, the size of the silicon block *L* was fixed to retain the amount of asymmetry and fabrication disorder. Although the number of arrays affecting the in-plane losses in quasi-BIC modes was changed, the whole array size (200 × 200 μm) could be significantly large to affect the *Q* factors. In practice, we did not find any difference in the peak intensities and the linewidths of the two peaks between metasurfaces with and without nanogaps, as can be found in Figure 6A and B. The refractive index sensitivities *S*_env_ were then obtained by linear fitting the peak shifts as a function of refractive indices (Figure 6C). The *S*_env_ for the metasurface with and without nanogaps were 317 nm/RIU and 117 nm/RIU, respectively. Therefore, the enhancement factor was 2.7, which agreed perfectly with the simulation. This result clearly shows that the localized electric field inside nanogaps largely interacted with the external medium. The slight mismatch with the simulation may be due to the increased mode overlap with the external medium caused by fabrication imperfections.

Table 1 summarizes the performance of our work. The FOM of the MD mode was 188 and 65.9 for the metasurface with and without nanogaps, respectively. It should be noted that the FOM can be potentially larger for metasurfaces with a smaller asymmetry because the *Q* factor becomes larger while maintaining the refractive index sensitivity (see Figure S4 Supporting Information for more details). For example, the *Q* factor for the metasurface with *s*/*L* of 1% was approximately 1200, corresponding to the FOM of 239. The FOM is among the highest reported in experimentally demonstrated dielectric metasurfaces, such as ring resonators with rectangular bars [28] (FOM ∼100), asymmetric double bars [39] (FOM ∼200), crescent resonators [19] (FOM < 50), and diatomic resonators [20] (FOM ∼ 70). A more detailed and comprehensive comparison of the sensing performance is also presented in Table 1. It is worth noting that the EQ mode in our work has a comparatively high refractive index sensitivity of 667 nm/RIU (detailed measurement results are shown in Figure S5 Supporting Information). Even though its *Q* factor and FOM were several times smaller than those of the MD mode, this finding provides application opportunities needing a higher bulk refractive index sensitivity offered by higher-order resonances of quasi-BICs. Some plasmonic structures, such as prism coupler-based surface plasmon resonance (SPR) sensors [40], fiber optic SPR sensors [41], and hyperbolic metamaterials [42], achieve even higher sensitivity *S*_env_ > 10^4^ nm/RIU. Although their *Q* factors are limited by their large optical losses, their FOM values are in the same order or slightly outperform our dielectric metasurfaces. But still, the mass-productive, precision-controlled fabrication capability of silicon-based metasurfaces using CMOS-compatible process is a great contrast to metal-based sensors.

**Table 1:** Comparison of experimentally measured sensing performance in different all-dielectric metasurfaces.

Unit structure		Material	*S*_env_ (nm/RIU)	*Q* factor	FOM	Ref.
Ring with a rectangular bar	w/o gaps	poly-Si	289	483	103	[28]
	w/ gaps		379	129	37	
Nanodisk		a-Si	227	20	5.4	[8]
Tilted nanobars		a-Si	263	144	∼44	[46]
Asymmetric double bars		a-Si	440 (simulation)	∼750	∼200	[39]
Crescent resonator		a-Si	326	∼120	<50	[19]
Diatomic resonator		a-Si	305	250	70.1	[20]
Tilted rectangular nanobars		a-Si	608 (local change)	∼100	46	[47]
Ring of asymmetric rod pairs		a-Si	–	>500	20	[48]
Rectangular block with a hole	MD, *s*/*L* = 1.0%, w/ gaps	c-Si	301	1233	239	This work
	MD, *s*/*L* = 2.0%, w/ gaps		317	923	188	
	MD, *s*/*L* = 2.0%, w/o gaps		117	895	65.9	
	EQ, *s*/*L* = 2.0%, w/ gaps		667	132	57.4	

Poly-Si: polycrystalline silicon; a-Si: amorphous silicon; c-Si: crystalline silicon.

For sensing applications based on their spectral shift, it is also important to increase the spectral SNR, as originally pointed out in Ref [43] and as discussed in more detail in Ref [44]. The higher the SNR_res_, the better the tracking resolution when the resonance peak is measured in real time because the variation between the spectral maxima and actual center position can be smaller. Here, we define the spectral SNR as [45]

(8) $$SNR_{\text{res}}=\frac{R_{\max}-R_{\min}}{\sigma_{\text{spectrum}}},$$

where *R*_max_ − *R*_min_ denotes the resonance amplitude determined from the Fano fitting and *σ*_spectrum_ corresponds to the standard deviation of the spectrum. As can be seen in Figure 6D, SNR_res_ increased with increasing asymmetry *s*/*L* because a more pronounced resonance peak intensity was obtained compared to the background noise level of the spectrum. On the contrary, SNR_res_ decreased with decreasing the asymmetry. Specifically, SNR_res_ were 67.7 and 18.6 for *s*/*L* of 2% and 1%, respectively. It implies the existence of a trade-off relationship between SNR_res_ and FOM. Therefore, it would be important not only to increase the FOM of the metasurface but also to appropriately select the intentional amount of asymmetry for sensing applications.

## 3 Conclusions

In conclusion, we have numerically and experimentally demonstrated all-dielectric metasurfaces with nanogaps (∼30 nm) that enhance FOM as refractometric sensors at the telecom wavelengths. We have successfully fabricated the metasurfaces using crystalline silicon-on-quartz wafers, showing quasi-BIC modes having both high *Q* factors over 1200 and high sensitivities over 300 nm/RIU. MD mode achieves a large interaction with the surrounding medium mainly inside nanogaps, making it robust against fabrication imperfections, compared with the EQ mode where a large part of the electric fields is localized outside the silicon blocks. Owing to the high *Q* factor enabled by quasi-BIC, we have demonstrated a high FOM of 239, among the highest in experimentally demonstrated all-dielectric metasurfaces. We have also pointed out the trade-off between the FOM and spectral SNR when the asymmetries of the metasurfaces are changed, which is an important property peculiar to photonic sensors based on quasi-BICs. Our results serve as highly sensitive refractometric sensing platforms and provide design strategies for highly sensitive biosensors based on quasi-BICs.

## 4 Methods

### 4.1 Simulation

The simulated spectra and electromagnetic field profiles were obtained by a commercial time-domain electromagnetic solver (Ansys Lumerical) based on the FDTD method. The mesh size was determined based on a default Lumerical conformal mesh technology supporting subcell features with a minimum mesh size of ∼14 nm. Only the nanogap region was overridden with a uniform mesh (Δ*x*, Δ*y*, Δ*z*) = (5 nm, 5 nm, 10 nm) for the simulation of electromagnetic field profiles to improve the spatial resolution. Additionally, a frequency-domain solver (COMSOL Multiphysics) based on the finite-element method (FEM) was used to calculate *Q* factors and conduct multipole decomposition. *Q* factors were calculated based on complex eigenfrequencies *f* by applying *Q* = Re(*f*)/2Im(*f*). In the simulation, Bloch (periodic) boundary conditions were set in the *x* and *y* directions for FDTD (FEM) and perfectly matched layers in the *z* direction for both simulations.

### 4.2 Fabrication

All-dielectric metasurfaces were fabricated on SOQ wafers (Shin-Etsu Chemical) with 200-nm crystalline silicon and 625-μm quartz layers. First, an SOQ wafer was cleaned with acetone and isopropyl alcohol (IPA) in an ultrasonic bath for 10 min, followed by O_2_ plasma ashing (PB-600Z, Yamato Scientific) for 3 min. An EB resist (ZEP520A, Zeon Chemicals) diluted by 1:1 with anisole (ZEP-A, Zeon Chemicals) was spin-coated onto the SOQ wafer at 6000 rpm for 60 s; after that, it was prebaked on a hotplate for 3 min at 180 °C. A conductive layer (ESPACER 300Z) was subsequently spin-coated at 2000 rpm for 60 s. The metasurface design (200 × 200 μm) was patterned using EB lithography (100 kV ELS-BODEN or 125 kV ELS-F125, Elionix) at the beam current of 500 pA. The sample was then developed by ZED-N50 for 1 min and ZMD-B for 30 s at room temperature. The written patterns were transferred into the 200-nm silicon layer using a Bosch process with SF_6_ and C_4_H_8_ gases (MUC-21 ASE-SRE, Sumitomo Precision Products). The remaining resist was removed by O_2_ plasma ashing for 20 min.

### 4.3 Angle-resolved transmittance measurements

Transmittance spectra were measured using a UV-NIS-NIR spectrometer equipped with the incident-angle-resolving unit (V7200, JASCO). Because the transmittance measurements require relatively large metasurfaces, a 2 × 2.5 mm metasurface was fabricated based on EB lithography. The metasurface was set to the equipment such that the plane of incidence of the *s*-polarized light was parallel to the longer axis of the metasurface (*x* direction). The transmittance spectra at different angles *θ* were acquired from 1.0 to 1.7 μm with an angle resolution of two degrees. The transmittance spectra were normalized to the transmittance without the metasurface sample.

### 4.4 Reflectance measurements

Reflectance spectra were measured using a home-built setup. A single mode tunable laser (TSL-510, Santec) with the wavelength range of 1.51–1.62 μm was used as an excitation source. The nanostructures and the laser spot were positioned by illuminating the sample with a halogen lamp from the bottom of the sample stage, and the transmission image was observed by an InGaAs camera (ARTCAM-031TNIR, Artlay). The collimated laser light passed through a 10× objective lens (M Plan Apo NIR NA = 0.26, Mitsutoyo) was focused on the metasurfaces, and the reflectance was measured by an InGaAs photodiode. Here, the polarization angles of the two polarizers (P1 and P2) were rotated by 90° to each other to fulfill the cross-polarized condition for eliminating the background noise while preserving the resonant scattering. The photodiode signal was measured with an oscilloscope (DPO3054, Tektronix) synchronized through the tunable laser with the sweep speed of 5 nm/s at 1 pm step. The reflectance spectra for refractometric sensing were measured in pure water filled in a custom-made PDMS microfluidic channel (Micro TAS engineering) by covering the PDMS channel with a height of 50 μm onto the fabricated metasurface sample. The different concentrations of IPA were injected through a silicone tube with an inner diameter of 1 mm and exchanged the solutions. The data were acquired on a proprietary LabVIEW (NI) program, where the reflectance spectra were normalized to the reflectance of an aluminum mirror.

## Supplementary Material

Supplementary Material Details

## Supplementary Material

The article contains supplementary material (https://doi.org/10.1515/nanoph-2022-0565).
