# Supplementary material for: Nanogap enhancement of the refractometric sensitivity at quasi-bound states in the continuum in all-dielectric metasurfaces
Source: Nanophotonics. 2023 Jan 3;12(1):99–109. doi: 10.1515/nanoph-2022-0565 (PMC11501443; doi:10.1515/nanoph-2022-0565)
Supplement: Supplementary file 1 — Supplementary Material Details [file j_nanoph-2022-0565_suppl.pdf]

Supporting Information for

Keisuke Watanabe*[1] and Masanobu Iwanaga[2]

Nanogap enhancement of the refractometric sensitivity at quasi-bound states in the continuum in all-dielectric metasurfaces

1. International Center for Young Scientists (ICYS), National Institute for Materials Science (NIMS), 1-1 Namiki, Tsukuba, Ibaraki 305-0044, Japan.
2. Research Center for Functional Materials, National Institute for Materials Science (NIMS), 1-1 Namiki, Tsukuba, Ibaraki 305-0044, Japan.

*E-mail: watanabe.keisuke@nims.go.jp

1 Polarization dependence

All-dielectric metasurfaces with nanogaps proposed in this work are polarization dependent. Figure S1 shows the FDTD simulated reflectance spectra when *s*-polarized plane waves are injected at different angles θ. The metasurface have nanogaps parallel to the *x*-direction and asymmetry s/L of 2%, where the airhole in each unit cell is shifted in the *y* direction. The superstrate is assumed to be water (n = 1.33). The simulation results show sharp quasi-BIC modes (EQ and MD) at normal incidence (θ = 0°) in the *x*-polarized light but not in *y*-polarized. On the other hand, both *x*- and *y*- polarized plane waves can couple with quasi-BIC mods when the incident angles are nonzero. We note that modes around 1.23 μm–1.25 μm are doubly degenerate modes, which have access to the far-field radiation in both *x* and *y* polarizations even at normal incidence.

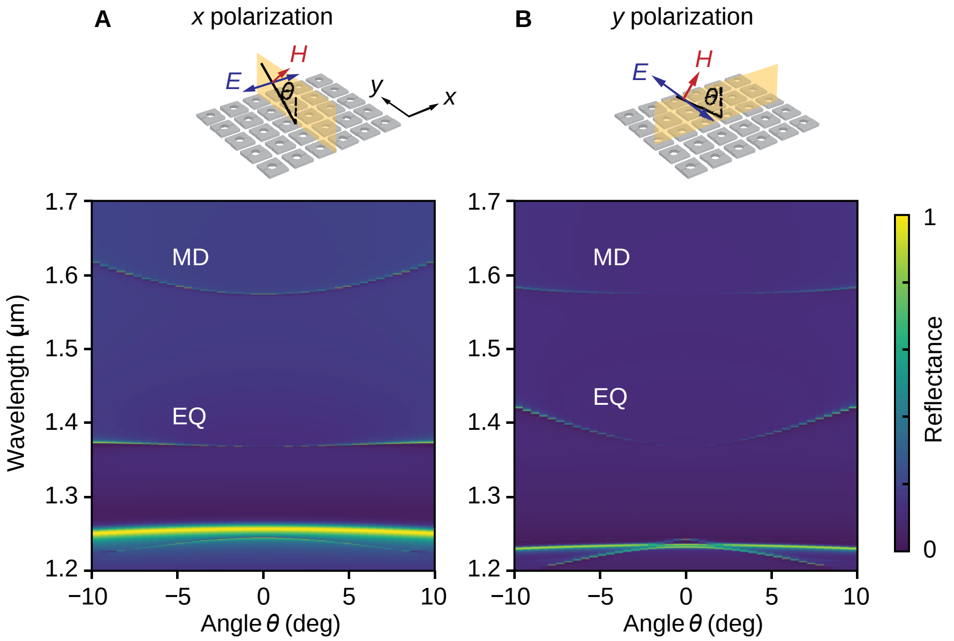

Figure S1: Simulated angle-resolved reflectance spectra of the metasurface with broken symmetry (s/L = 2%) when the incident plane waves have (A) *x* polarization and (B) *y* polarization. The superstrate is assumed to be water (n = 1.33).

2 Measured spectra of the EQ mode

Here we show from the experimentally measured spectra, obtained by angle-resolved transmittance measurements and reflectance measurements at normal incidence, that the linewidths of the EQ mode were wider than that of the MD mode. Figure S2 shows the cross-cut spectra of the metasurface without broken symmetry (s/L = 0%) measured in air at the incident angles θ of 16°, 22°, and 30°. With the increase in the angle of incidence θ, the EQ and MD modes can be accessible from the free space excitation. At θ = 16°, the EQ mode had a smaller dip of the transmittance and slightly wider linewidth than those of the MD mode. The results for θ = 22° and 30° show that the linewidths of the EQ mode increased, while the MD mode did not experience much change in the linewidths. Figure S3 shows measured reflectance spectra at normal incidence of the metasurfaces with broken symmetry s/L of 2%. The period P of the metasurface was expanded (P = 1000 nm) while fixing the size of the unit structure L (it is presumed that the effect of the fabrication imperfections is approximately the same as the MD mode) to reside the resonance wavelength of the EQ mode in the sweep wavelength range of the tunable laser. Similarly, the resonance linewidth of the EQ mode was several times larger than that of the MD mode. In summary, the linewidths of the EQ mode were a few times larger than those of the MD mode in both measurements with different incident angles and asymmetries. The calculation shown in Figure 1D indicates that the Q factors in the EQ mode are slightly larger than those in the MD mode, unlike our experiment. As we have stated in the main text, these results can be understood from the fact that the electric fields of the EQ mode are localized outside the silicon blocks and the Q factors are likely to be affected by fabrication imperfections such as local deformations and rounded corners.

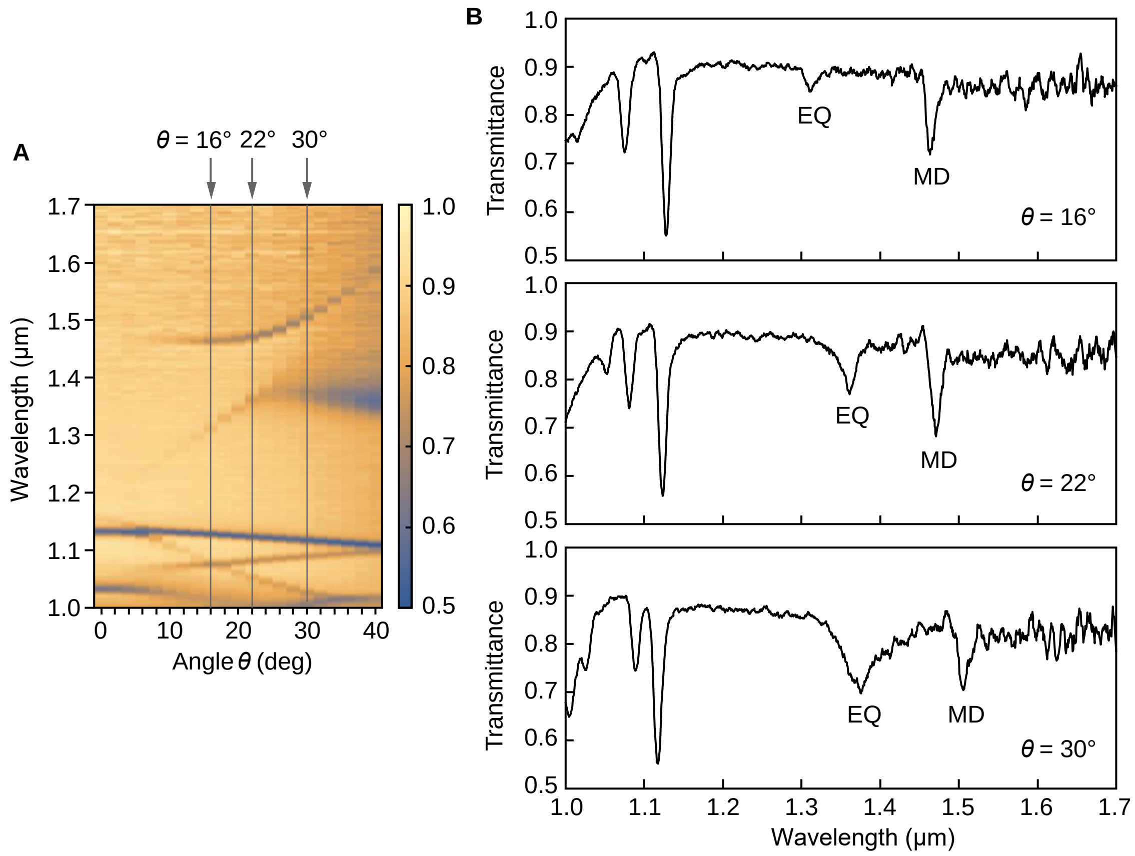

Figure S2: Angle-resolved transmittance spectra of the metasurfaces without broken symmetry (s/L = 0%) measured in air. An s-polarized light with a wave vector along the y direction was used. (A) Measured contour map of the transmittance. (B) Spectra measured at the incident angle θ of 16°, 22°, and 30° from top to bottom, respectively.

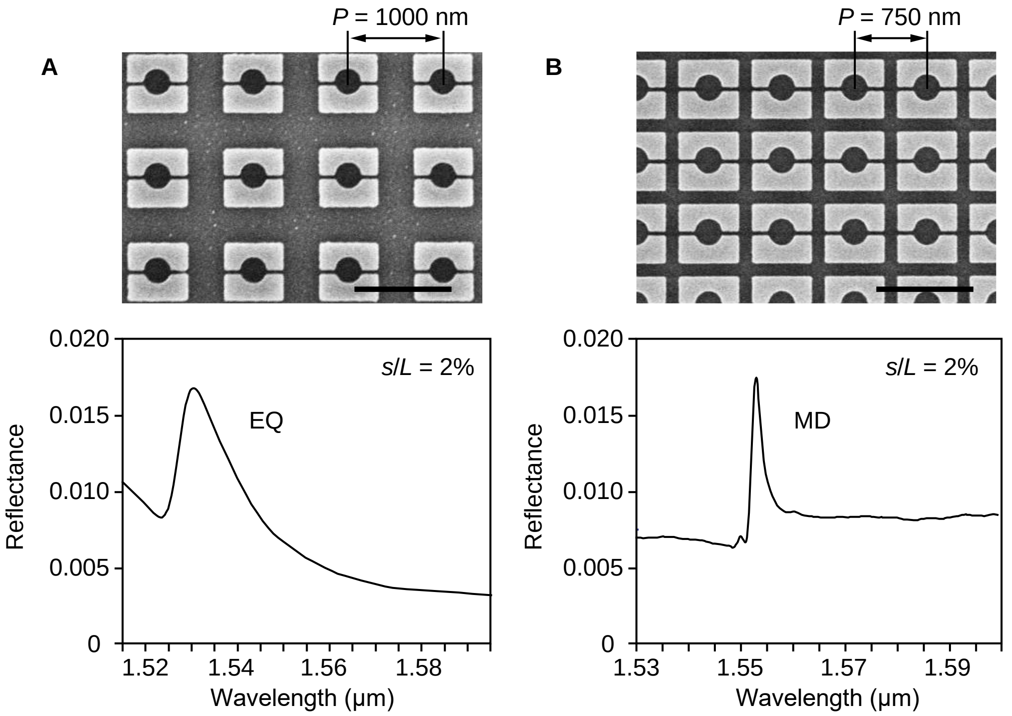

Figure S3: SEM images (upper) and the measured reflectance spectra (lower) of the metasurfaces with broken symmetry *s*/*L* of 2%, fabricated for measuring (A) EQ (*P* = 1000 nm) and (B) MD (*P* = 750 nm) modes. Scale bars represent 1 μm. The measurements were conducted in water.

3 Comparison of FOM with and without nanogaps

Figure S4 compares the refractive index sensitivity S_{env} and FOM with and without nanogaps. The index sensitivities remained approximately the same in different degrees of asymmetry *s*/*L*, meaning that the shift of the airhole in each unit cell did not change the confinement factor of the MD mode outside the silicon blocks. The averages of the S_{env} with and without nanogaps were 320 nm/RIU and 118 nm/RIU, respectively. On the contrary, the FOMs decreased with increasing asymmetries *s*/*L* because the larger *s*/*L* decreased the *Q* factors. But still, the FOMs were larger for the metasurfaces with nanogaps than those without nanogaps because there were little differences in the *Q* factors in both cases at a given asymmetry.

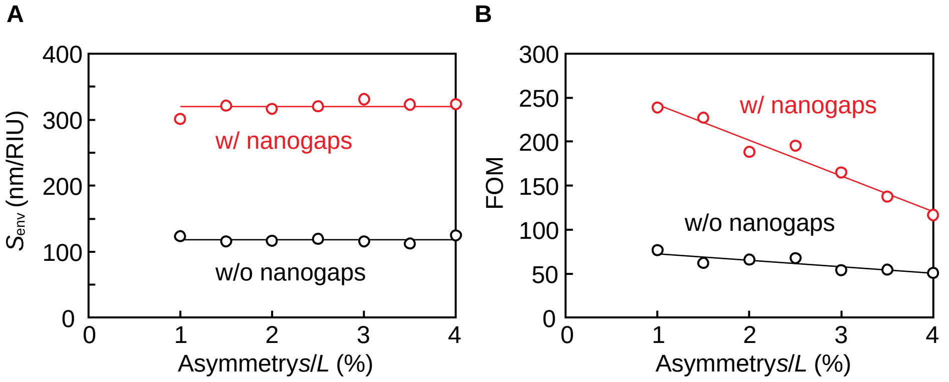

Figure S4: Experimentally obtained (A) environmental refractive index sensitivity S_{env} and (B) FOM with (*g* ~33 nm) and without nanogaps for the MD mode.

4 FOM of the EQ mode

Although the EQ mode is more susceptible to fabrication imperfections and has *Q* factors several times lower than the MD mode, the relatively large refractive index sensitivity is noteworthy. Figure S5 shows the measurement results of the refractive index sensitivity of the EQ mode. The sensitivities for the metasurfaces with and without nanogaps were 667 nm/RIU and 638 nm/RIU, respectively. The slightly larger sensitivity with nanogaps than that without nanogaps was consistent with the simulation, but both sensitivities were unexpectedly large. We attribute this result to the increased confinement factor of the EQ mode outside the silicon blocks resulting from the fabrication imperfections. The refractive index sensitivity of the EQ mode with nanogaps (667 nm/RIU) corresponds to twice that of the MD mode (317 nm/RIU) and is among the highest in similar all-dielectric metasurfaces.

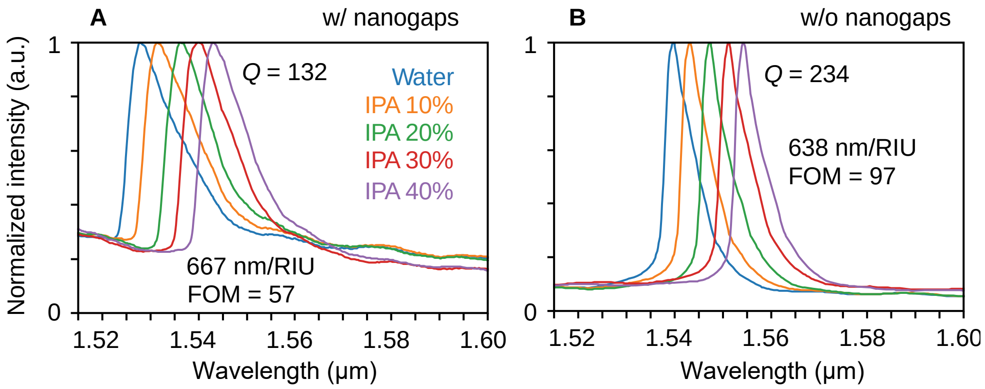

Figure S5: Measurement results of the refractive index sensitivity of the EQ mode. (A),(B) Normalized reflectance spectra measured in different concentrations of IPA for the metasurfaces (s/L = 2%) with and without nanogaps, respectively.
